# Association of high serum vitamin D concentrations with active pulmonary TB in an HIV co-endemic setting, Harare, Zimbabwe

**DOI:** 10.1186/s12879-017-2243-x

**Published:** 2017-02-13

**Authors:** Cuthbert Musarurwa, Lynn Sodai Zijenah, Doreen Zvipo Duri, Kudzie Mateveke-Dangaiso, Kudakwashe Mhandire, Maria Mary Chipiti, Marshall Wesley Munjoma, Witmore Bayayi Mujaji

**Affiliations:** 1 Department of Chemical Pathology, University of Zimbabwe, College of Health Sciences, P.O. Box A178, Avondale, Harare Zimbabwe; 2 Department of Immunology, University of Zimbabwe, College of Health Sciences, P.O. Box A178, Avondale, Harare Zimbabwe; 3 Research Support Centre, University of Zimbabwe, College of Health Sciences, P.O. Box A178, Avondale, Harare Zimbabwe; 4 Department of Obstetrics and Gynaecology, University of Zimbabwe, College of Health Sciences, P.O. Box A178, Avondale, Harare Zimbabwe

**Keywords:** Vitamin D deficiency, Pulmonary tuberculosis, HIV status, Harare, Zimbabwe

## Abstract

**Background:**

There is paucity data on the association of vitamin D deficiency (VDD) and active tuberculosis (TB) in southern Africa where the human immunodeficiency virus (HIV) is co-endemic. We examined the association of serum vitamin D concentrations with active pulmonary tuberculosis (PTB) in HIV-infected (*n* = 284) and uninfected (*n* = 267) Black Zimbabweans, in Harare, Zimbabwe.

**Methods:**

We conducted a cross-sectional study of 551 participants comprising 145 HIV^+^/PTB ^+^, 139 HIV^+^/PTB^−^, 134 HIV^−^/PTB^+^ and 133 HIV^−^/PTB^−^. PTB status was confirmed using sputum by culture, or smear microscopy, or GeneXpert MTB/RIF. Serum 25-hydroxyvitamin D (25(OH)D) concentrations were measured using a competitive chemiluminescent immunoassay prior to commencement of anti-TB treatment.

**Results:**

In all four groups, the median vitamin D concentrations were above the 20 ng/ml cut off for VDD. However, the median vitamin D concentrations in all the four groups were below the cut off for vitamin D sufficiency ≥30 ng/ml. The median vitamin D concentrations were significantly higher in PTB^+^ cases; 24.2 ng/ml (IQR: 18.8–32.0) compared to PTB^−^ controls 20.9 ng/ml (IQR: 17.1–26.9), *p* < 0.0001 regardless of HIV status. The HIV^+^/PTB^+^ group had the highest median vitamin D concentration (25.3 (IQR: 18.0–33.7 ng/ml) whilst the HIV^+^/PTB^−^ group had the lowest; 20.4 ng/ml (IQR: 14.6–26.9), *p* = 0.0003. Vitamin D concentration <30 ng/ml was associated with 43% lower odds of being PTB^+^ OR 0.57 (95% CI 0.35–0.89).

**Conclusions:**

Our results are not in agreement with the generally accepted hypothesis that VDD is associated with active PTB. To the contrary our results showed an association of higher vitamin D concentrations with active TB irrespective of HIV status. Although findings from the available pool of case control studies remain inconsistent, the results from the current study provide further rationale for larger-scale, prospectively designed studies to evaluate whether sufficient vitamin D concentrations do indeed precede the development of active PTB in our setting.

## Background

Zimbabwe, a country ranked 17^th^ out of 22 high burden countries that together contribute 80% of the global tuberculosis (TB) burden, had a TB prevalence of 409/100 000 in 2013 [1]. The resurgence of the TB epidemic in Zimbabwe is fuelled by HIV with ~80% co infection rates [1].

Susceptibility to TB is associated with host immune response which in turn may be influenced by environmental and genetic factors or interactions between the two [2, 3]. Vitamin D, an immunomodulatory effector via the vitamin D receptor, has been reported to be critical in inducing antimycobacterial activity by inhibiting the growth of *Mycobacterium tuberculosis* (MTB) and up-regulating innate host responses [4–6]. Evidence of this was derived from in vitro studies which demonstrated that vitamin D metabolites regulate the expression of cathelicidin (LL-37), an endogenous antimycobacterial peptide in cultured macrophages [7–10].

There is paucity of data on association of serum vitamin D concentrations with TB in southern Africa where HIV is co-endemic. We thus conducted a cross-sectional study to measure the concentrations of serum vitamin D and to examine their association with active pulmonary TB (PTB) in HIV-infected and uninfected Black Zimbabweans in Harare, Zimbabwe.

## Methods

### Study setting and design

Harare is located at latitude 17°55′S and longitude 31°7′E and at an altitude of 1480 m. The study participants were all dark skinned and unveiled, with faces and arms regularly exposed to the sun.

We used stratified sampling to enrol consecutive consenting participants into four participant groups: HIV/TB co-infected (HIV^+^/PTB^+^), HIV infected/PTB negative (HIV^+^/PTB^−^), HIV uninfected/PTB positive (HIV^−^/PTB^+^) and HIV/TB uninfected (HIV^−^/PTB^−^). HIV infected participants were under routine care as specified by prevailing national guidelines whereas PTB^+^ participants were TB treatment naïve prior to measurement of serum vitamin D concentrations.

### Study participants

**Fig. 1:**
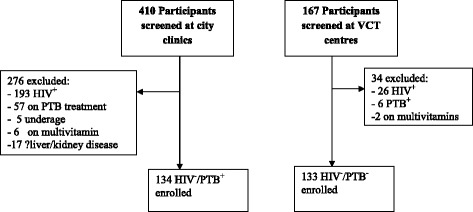
Participants Enrolment Chart. HIV = Human immunodeficiency virus; HIV^+^ = HIV positive; HIV^−^ = HIV negative; PTB = Pulmonary tuberculosis; PTB^+^ = PTB positive; PTB^−^ = PTB negative

HIV^−^ participants aged >18 years were recruited from City of Harare Clinics. HIV^−^/PTB ^−^ participants were healthcare workers and attendees at the institutional voluntary HIV testing and counseling centres with documented HIV and PTB negative status (Fig. 1). Similarly, HIV^−^/PTB^+^ enrolled at the same centres had documented HIV and PTB status. PTB infection was confirmed on sputum by culture, smear microscopy or by Xpert MTB/RIF ® (Cepheid Sunnyvale, United States). At enrolment, sociodemographic characteristics and clinical variables were assessed and recorded by completion of a questionnaire for all consenting participants. Thereafter, 5mls venous blood was collected by venipuncture from which serum was harvested by centrifugation at 3000 rpm for 5 min and stored at −80 °C until subsequent measurements of vitamin D concentrations.

Stored serum specimens for the HIV^+^/PTB^+^ and HIV^+^/PTB^−^ groups were obtained from participants who were enrolled in a randomised controlled trial (RCT) entitled ‘A randomized controlled trial to evaluate the impact of using a point-of-care urine lipoarabinomannan (LAM) strip test for TB diagnosis amongst hospitalized HIV-infected patients in resource-poor settings (RCT LAM)’. The RCT LAM was enrolling patients at the same period as recruitment was taking place from the city of Harare clinics, the institutional voluntary HIV testing and counseling centres in Harare. Details of the RCT are described elsewhere [11, 12]. Appropriate demographic and laboratory data for these two groups of participants were abstracted from the RCT LAM database. All such data and specimens were anonymized before access was allowed to the current investigators.

### Case definition

PTB cases either HIV-infected or –uninfected, were sputum smear microscopy or culture (using mycobacterial growth indicator tube) or GeneXpert MTB/RIF positive.

### Control definition

Controls were PTB culture negative and either HIV-infected or uninfected.

### Bacteriological testing

At enrolment, at least three sputum specimens were collected from each participant for same day testing (MTB/RIF, or microscopy). One sputum specimen was submitted for culture.

Sputum smear microscopy using a fluorescence microscope was done with auramine stain for screening and confirmation of auramine positive smear was with Ziehl-Neelsen (Z-N) stain.

The Xpert MBT/RIF assay was performed as previously described [13]. Briefly, the sample reagent was added in a volume twice that of the untreated sputum and incubated for 15 min. Two millimetres of the processed sputum was then transferred to the MTB/RIF assay catridge and then inserted into the Gene Xpert instrument.

The Mycobacteria Growth Indicator Tube (MGIT BD Microbiology Systems, Cockeysville, MD, USA) culture was performed on sputum decontaminated using 4% NaOH. Suspected positive cultures were confirmed using Z-N staining. MPT64 antigen detection was used for speciation of MGIT positive cultures and by growth at different temperatures if antigen detection was negative [14].

### Vitamin D assays

Serum 25(OH)D was measured as a marker of vitamin D status using a fully automated competitive chemiluminescent immunoassay analyser (Maglumi 2000 Snibe Co. Ltd Shenzhen, 518057 China). The assay was carried out in accordance with the manufacturer’s instructions. Serum specimens were thawed only once for the analysis cycle. Between-run precision coefficients of variation for the assay ranged from 6.04 to 6.25% and within run precision coefficients of variation ranged from 3.01 to 3.45%.

Serum 25(OH)D concentrations are generally accepted as being reflective of the overall vitamin D status because of a longer half-life of approximately 15 days compared to 15 h for the active form [15, 16]. Thus in this write up, serum vitamin D concentration is used synonymously with serum 25(OH)D concentration. Vitamin D deficiency (VDD) was defined as serum 25(OH)D concentration <20 ng/mL, vitamin D insufficiency as serum 25(OH)D concentration ≥20–29 ng/mL, and sufficient vitamin D status as serum 25(OH)D ≥30 ng/ml [17, 18]. Vitamin D concentrations below 10 ng/ml were classified as severely vitamin D deficient [19].

### Ethics statement

The study protocol was approved by the Medical Research Council of Zimbabwe (MRCZ/A/1906). All the participants enrolled from the Harare City Clinics gave their written informed consent to participate in the study. All the participants from the RCT LAM consented to use of their leftover archived specimens and data in future TB related studies.

### Statistical analyses

All statistical analyses were conducted using the STATA statistical software package (version 13.0; Stata Corporation, College Station, Texas, USA) and GraphPad Prism (version 5; GraphPad Prism Inc, San Diego, CA, USA). Data were summarised by count and proportion (%) and median and interquartile range (IQR) for non-normally distributed variables or means ± standard deviations for normally distributed data. Chi-square test was used to test for differences in proportions. Differences between groups of continuous normally distributed data were tested using independent samples *t*-test and ANOVA whilst the non-parametric Mann-Whitney or Kruskal Wallis tests were used respectively for non-normal data. Dunn’s test for multiple cross group rank sum comparisons was used to compare median vitamin D levels by HIV/PTB status. For all statistical comparisons α was set at 0.05. Co-variates of VDD were determined using logistic regression as was the effect of vitamin D status on PTB status. Logistic regression was also used to determine the association between PTB and vitamin D deficiency, insufficiency, optimal and severe vitamin D deficiency. Odds ratios and their 95% confidence intervals were reported for all such cases.

## Results

### Study population

The clinico-demographic data of the 551 study participants stratified into four groups HIV^+^/PTB^+^ (*n* = 145), HIV^+^/PTB^−^ (*n* = 139), HIV^−^/PTB^+^ (*n* = 134) and HIV^−^/PTB^−^ (*n* = 133) are presented in Table 1.

**Table 1:** Participant demographics

Variable	All *n* = 551	HIV^+^/PTB^+^ *n* = 145	HIV^+^/PTB^−^ *n* = 139	HIV^−^/PTB^+^ *n* = 134	HIV^−^/PTB^−^ *n* = 133	*p* value
Age in years; mean(SD)	38.5 (12.3)	38.6(9.0)	40.5(12.6)	39.7(16.5)	35.1(8.9)	0.0017
Males n (%)	272 (51)	63(48)	70(50)	84 (64)	55 (42)	^*^

^*^Significant difference between HIV^+^/PTB^−^ and HIV^−^/PTB^+^ *p* = 0.02 and significant difference HIV^−^/PTB^+^ and HIV^−^/PTB^−^

The mean age of the study participants was 38.5 years (SD12.3). Participants in the HIV^−^/PTB^−^ group were statistically significantly younger than those in the HIV^+^/TB^−^ group; *p* = 0.002 and the HIV^−^/TB^+^ group *p* = 0.016 (Table 1). The proportion of males in the HIV^−^/TB^+^ (64%) was significantly higher that of the HIV^−^/TB^−^ group (42%), *p* = 0.0003.

The vitamin D concentrations and status of the four study groups are shown in Table 2.

**Table 2:** Vitamin D concentrations status stratified by HIV/TB status

	All *n* = 551	HIV^+^/PTB^+^ *n* = 145	HIV^+^/PTB^−^ *n* = 139	HIV^−^/PTB^+^ *n* = 134	HIV^−^/PTB^−^ *n* = 133
Vitamin D ng/ml median (IQR)	22.5 (17.7–29.3)	25.3 (18.0–33.7)	20.4 (14.6–26.9)	24.0 (19.5–29.6)	21.6 (18.3–26.5)
Vitamin D status					
Vitamin D deficient (n%) (<20 ng/ml)	205(37.2)	49(33.8)	67(48.2)	38(28.4)	51(38.4)
Severe vitamin D deficiency (n%) (<10 ng/ml)	6(1.1)^a^	0	5(3.6)^a^	1(0.8)^a^	0
Vitamin D insufficient (n%) (≥20 < 30 ng/ml)	218(39.6)	46(31.7)	42(30.2)	64(47.9)	66(49.6)
Vitamin D sufficient (n%) (≥30 ng/ml)	128(23.2)	50(34.5)	30(21.6)	32(23.9)	16(12.0)

^a^Severe vitamin D deficient participants also included in vitamin D deficient participant frequency

**Fig. 2:**
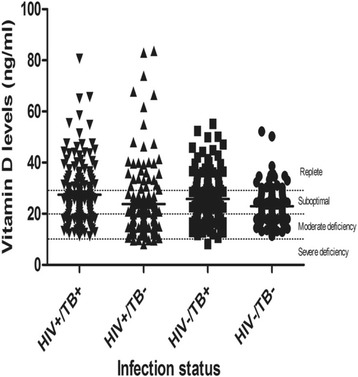
Serum vitamin D concentrations in HIV^+^/PTB^+^ (*n* = 145), HIV^+^/PTB^−^ (*n* = 139), HIV^−^/TB^+^ (*n* = 134), and HIV^−^/PTB^−^ (*n* =133). Serum vitamin D: 25-hydroxyvitamin D; TB: Pulmonary Tuberculosis; HIV = human immunodeficiency virus; +: Positive; −: Negative. The *solid horizontal lines* () indicate the median vitamin D level for each. The *broken horizontal lines* (……) demarcate the cutoff points for each vitamin D status category

The overall median serum vitamin D concentration was 22.5 ng/ml (IQR 17.7–29.3) (Table 2). Median serum vitamin D concentrations were statistically significantly different by HIV/PTB infection status; *p* = 0.0001. The HIV^+^/PTB^+^ group had the highest median serum vitamin D concentration 25.3 ng/ml (IQR 18.0–33.7) whilst the HIV^+^/PTB^−^ group the lowest 20.4 (IQR14.6–26.9) ng/ml. The median serum vitamin D concentration was statistically significantly higher for the HIV^+^/PTB^+^ group versus the HIV^+^/PTB^−^ group (*p* < 0.0001) and the HIV^−^/PTB^−^ group (*p* = 0.008). The median serum vitamin D concentration was also statistically significant higher for the HIV^−^/PTB^+^ group versus the HIV^−^/PTB^−^ (*p* = 0.02) and the HIV^+^/PTB^−^ group (*p* = <0.001). However there were no statistically significant differences in median vitamin D concentrations between the HIV^+^/PTB^+^ and HIV^−^/PTB^+^ groups *p* = 0.41 and between the HIV^+^PTB^−^ and HIV^−^/PTB^−^ groups *p* = 0.06. None of the four study groups had median vitamin D concentrations above the optimum cut off point of 30 ng/ml (Fig. 2).

The proportions of individuals in each of the vitamin D status strata were significantly different across the 4 HIV/PTB participant groups (*p* < 0.001). (Table 2) The HIV^+^/PTB^−^ group had the highest proportion (48%) of vitamin D deficient participants whilst the HIV^−^/PTB^+^ group had the lowest proportion (28%); *p* = 0.0008. The HIV^+^/PTB^+^ group had the highest proportion (35%) of individuals with sufficient vitamin D concentration (≥30 ng/ml) whilst the HIV^−^/PTB^−^ had the lowest proportion (12%); *p* < 0.0001.

### Correlates of serum vitamin D levels

The association between serum vitamin D status was further analysed by comparing the median serum vitamin D concentrations by age, gender, HIV and PTB status (Table 3). Univariate logistic regression was conducted to ascertain determinants of serum vitamin D status. All univariate logistic regression factors with a *p*-value that was less than 0.25 were included in the multiple logistic regression model.

**Table 3:** Co-variates of serum vitamin D status

Variable	Serum Vitamin D ng/ml Median(IQR)	*p*-value	Odds ratio 95%CI
Gender *n* = 533			
1. Male *n* = 272	21.98 (16.4–28.5)	0.017	Referent
2. Female	22.83 (18.5–29.9)		0.8 (0.5–1.2)
Age *n* = 550			
1. ≤50 years *n* = 452	22.63 (17.7–29.4)	0.409	Referent
2. >50 years	22.05 (17.6–28.8)		0.8 (0.5–1.3)
TB status *n* = 550			
1. PTB positive *n* = 279	24.21 (18.8–32.0)	<0.001	0.57 (0.35–0.89^a^
2. PTB negative	20.91 (17.1–26.9)		
HIV status *n* = 550			
Infected *n* = 283	22.14 (16.4–31.5)	0.394	0.38 (0.24–0.60)^a^ 1.86 (1.26–2.75)^b^
Uninfected	22.63 (18.9–28.4)		

^a^The odds ratio referent was hypovitaminosis D <30 ng/ml compared to optimal vitamin D concentration >30 ng/ml

^b^Referent was serum vitamin D <20 ng/ml

Disregarding the HIV status, PTB^+^ participants had statistically significantly higher median serum vitamin D concentration compared to PTB^−^ participants (*p* < 0.0001). Median serum vitamin D concentrations were also statistically significantly higher in females compared to males (*p* = 0.017) but did not differ significantly by HIV status or age >50 years. Gender was not a significant correlate of vitamin D status in both univariate and multivariate logistic analysis but age > 50 years in multivariate analysis was a predictor of vitamin D deficiency with adjusted OR 1.05 (95%CI 1.01–1.1). Participants with vitamin D concentrations <30 ng/ml were 43% less likely to be PTB^+^; OR 0.57 (95% CI 0.35–0.89) and 62% less likely to be HIV^+^ compared to those with sufficient vitamin D concentrations. VDD OR 0.75 (95%CI 0.51–1.10) and severe VDD OR 0.26 (95%CI 0.03–2.29) were not associated with PTB positivity.

## Discussion

Our study has some intriguing findings. Firstly, in our study population the prevalence of vitamin D deficiency was 37%. Secondly, the median serum vitamin D concentration in all the four groups was above the cut off level defining VDD (<20 ng/ml), ironically with highest medians being in the PTB^+^ groups regardless of HIV status compared to the PTB^−^ groups. Thirdly, and paradoxically, the PTB^+^ groups irrespective of HIV status had the lowest proportions of participants with serum VDD when compared to the PTB^−^ groups. Fourthly, and conversely, the PTB^+^ groups had the highest proportions of participants with sufficient (≥30 ng/ml) vitamin D concentrations compared to the PTB^−^ controls, implying that active PTB is associated with relatively, higher concentrations of vitamin D. In support of this is the observation that the double negative control group, (HIV^−^/TB^−^) had the highest proportion of participants with insufficient serum vitamin D concentrations. Fifthly and similarly, this group had the lowest proportion of participants with sufficient vitamin D concentrations. Indeed in a multivariate analysis insufficient concentrations of vitamin D were associated with a lower risk of being PTB^+^. Sixthly, age >50 years was a predictor of vitamin D deficiency.

VDD has been associated with increased risk of several infectious and metabolic diseases [3, 20, 21]. In vitro studies further indicate that physiological concentrations of 1,25 dihyroxycholecalciferol (1,25D3) inhibit both HIV and *M. tuberculosis* replication in human macrophages via an autophagy and cathelicidin dependent mechanism [10]. The in-vivo association between vitamin D status and tuberculosis however remains contentious [16, 22–24].

To our knowledge, only three other studies in sub Saharan Africa have examined the association of vitamin D status with TB in a HIV endemic setting [16, 22, 25]. Our observations of higher median vitamin D concentrations, lower proportions of participants with vitamin D deficiency and insufficiency in the PTB^+^/HIV^+^ groups regardless of HIV status are similar to those reported in a cross-sectional study conducted in Tanzania [25]. In sharp contrast to our results, the observational study in Cape Town, South Africa, with four groups of study participants similar to our study (although their study distinguished latent from active TB), reported a high prevalence (62.7%) of VDD (<20 ng/ml) in all the four groups [22]. Active TB was associated with VDD in both HIV-infected and uninfected participants with the association being stronger in the former group. Optimal/sufficient (>30 ng/ml) serum vitamin D concentrations were associated with active TB in HIV-infected but not in the HIV-uninfected participants. The study also reported a reciprocal seasonal variation in vitamin D status and tuberculosis notifications. We did not investigate this aspect in Zimbabwe and we do not know if the seasonal variation may partially explain the differences in the results from our study. A prospective observational study with similar study participants groups to ours (but a smaller cohort), conducted in Uganda, reported low prevalence of VDD with more than 50% of the participants having optimal vitamin D concentrations in all the four groups [16]. There were no significant differences in vitamin D status or the median vitamin D concentrations in the four groups. The observed differences between the two studies could be explained by the proximity of Uganda to the equator and the likely differences in the solar zenith angle that would enable an individual close to the equator to synthesize vitamin D more efficiently [26].

Although our findings are discordant with the generally accepted hypothesis that VDD is associated with PTB [22, 23], they are biologically plausible. PTB infection infamously known as ‘the consumption’ [27] in the past, could lead to higher vitamin D levels by two mechanisms. Adipose tissue is a reservoir of vitamin D [28] and wasting associated with PTB could result in increased release of sequestered vitamin D into circulation. Higher vitamin levels reported by others, in participants with body mass index (BMI) ≤ 18.5 compared to those of higher BMI lend further support to our speculation [22, 25]. On the other hand, MTB induces expression of 1-α-hydroxylase (CYP27B1) in macrophages thus mediating local conversion of 25(OH)D to 1,25D3. This induction enhances the ability of macrophages to restrict mycobacterial growth [6, 10]. Heightened immune activation in HIV^+^/PTB^+^ participants might therefore lead to increased consumption of 25(OH) D and its further mobilisation from adipose tissue into circulation.

Several other studies to examine vitamin D status and association with PTB have been conducted globally albeit with conflicting results. In general, studies performed in African [19, 22, 29, 30] and Caucasian populations [31] have reported lower vitamin D concentrations in TB patients than in controls while the results from Asia are variable. There were no significant differences in median vitamin D concentrations between TB cases and controls in Indonesia [32] China [33], South Korea [34] and Afghanistan [35]. In contrast, in a recent study conducted in Vietnam, vitamin D insufficiency was more prevalent in males with TB than in controls [36].

The geographical variation of vitamin D status and association with TB may be attributable to differences in cultural behaviours, exposure to sunshine, dietary intake of vitamin D, and genetic factors. A study of Asians in London reported that genetic polymorphisms in the vitamin D receptor gene can result in differing susceptibility to VDD [23].

The conflicting results on the association of vitamin D deficiency with an increased risk of PTB may also be attributable to the differing criteria for VDD. In some studies, a concentration of 25-(OH)D3 < 20 ng/ml was used as a cut off [19, 22, 23], while other studies used a concentration of 25-(OH)D3 of <25 nmol/L [34, 36]. Some studies used other criteria such as 25–50 nmol/L [16]. The criteria for other commonly used terminologies such as vitamin D insufficiency, severe insufficiency and sufficient and their association with TB also vary across the studies [16, 19, 22, 23, 35] We selected the vitamin D deficiency, insufficiency and sufficiency thresholds as widely recommended in other studies [17, 18]. Mao et al*.* raise a point of caution on the possibility that the cut off points as suggested by the Institute of Medicine, which are based on skeletal health for a predominantly Caucasian population, may not be ideal for other races in other settings [37]. It might therefore be prudent to establish local reference intervals for different settings and racial groups.

### Limitations

A major limitation of the current analysis is the cross-sectional design which precludes causal inferences to be made on the observed associations due to lack of temporality and the possibility of reverse causality. The unmatched nature of the comparison groups in this cross-sectional design probably also impedes drawing of stronger conclusions. Other limitations include lack of data on sunshine exposure and dietary habits. Despite these, the study had a major advantage of a fairly large sample size and also included HIV^+^ and HIV^−^ comparison groups unlike some similarly designed studies that were underpowered or did not include PTB^−^ comparison groups.

## Conclusions

In our study vitamin D concentration <30/ng/ml, were associated with lower odds of being PTB^+^. Paradoxically, the PTB^+^ groups regardless of HIV status not only had the highest median serum vitamin D concentrations but also the highest proportion of participants with sufficient vitamin D concentration (≥30 ng/ml) implying that higher median and optimal vitamin D concentrations are associated with active PTB. We recommend prospective studies to evaluate whether sufficient vitamin D concentrations precede the development of active PTB.

## Abbreviations

25(OH)D: 25-hydroxyvitamin D
ANOVA: Analysis of variance
BMI: Body mass index
HIV: Human immunodeficiency virus
IQR: Interquartile range
LAM: Lipoarabinomannan
MRCZ: Medical research council of Zimbabwe
MTB: *Mycobacterium tuberculosis*
OR: Odds ratio
PTB: Pulmonary tuberculosis
RCT: Randomised controlled trial
SD: Standard deviation
TB: Tuberculosis
VDD: Vitamin D deficiency
Xpert MTB RIF: GeneXpert *Mycobacteria tuberculosis* Rifampicin
